# Evaluation of objective and subjective binocular ocular refraction with looking in type

**DOI:** 10.1186/s12886-024-03449-y

**Published:** 2024-04-16

**Authors:** Megumi Fukushima, Masakazu Hirota, Takafumi Yukimori, Akio Hayashi, Yoko Hirohara, Makoto Saika, Kumiko Matsuoka

**Affiliations:** 1 Division of Orthoptics, Graduate School of Medical Care and Technology, Teikyo University, Itabashi, Tokyo Japan; 2 Department of Orthoptics, Faculty of Medical Technology, Teikyo University, 2-11-1 Kaga, Itabashi, 173-8605 Itabashi, Tokyo Japan; 3 Department of Ophthalmology, School of Medicine, Teikyo University, Itabashi, Tokyo Japan; 4 Topcon Corporation, Itabashi, Tokyo Japan

**Keywords:** Ocular refraction, Refractive error, Astigmatism, Autorefractometer, Visual acuity

## Abstract

**Background:**

This study aimed to compare the results of the Chronos binocular/monocular refraction system, that measures objective and subjective ocular refraction in one unit, to objective findings obtained from a conventional autorefractometer and a conventional subjective ocular refraction using a trial-frame in real space.

**Methods:**

Twenty-eight healthy volunteers (21.2 ± 1.5 years old) were included in this study. Objective ocular refraction was measured using two tests: the Chronos binocular/monocular refraction system under binocular conditions and a conventional autorefractometer under monocular conditions. Subjective ocular refraction was measured using three tests: Chronos binocular/monocular refraction system under binocular, monocular conditions, and trial-frame in the real space under monocular conditions. The measurement distance was set to 5.0 m for each test. All ocular refractions were converted into spherical equivalents (SEs).

**Results:**

The objective SE was significantly more negative with Chronos binocular/monocular refraction system under binocular condition (− 4.08 ± 2.76 D) than with the conventional autorefractometer under monocular condition (− 3.85 ± 2.66 D) (*P* = 0.002). Although, the subjective SE was significantly more negative with Chronos binocular/monocular refraction system under binocular condition (− 3.55 ± 2.67 D) than with the trial-frame in the real space under monocular condition (− 3.33 ± 2.75 D) (*P* = 0.002), Chronos binocular/monocular refraction system under monocular condition (− 3.17 ± 2.57 D) was not significantly different from that in trial-frame in real space under monocular condition (*P* = 0.33).

**Conclusion:**

These findings suggest that the Chronos binocular/monocular refraction system, which can complete both objective and subjective ocular refraction tests in a single unit, is suitable for screening ocular refraction, although it produces slightly more myopic results. Furthermore, subjective ocular refraction testing accuracy in Chronos binocular/monocular refraction system can be equivalent to trial-frame in real-space testing by switching from binocular to monocular condition.

## Background

Autorefractometers are one of the most commonly used tools in eye care practice [1]. Almost all eye care practitioners base their subjective ocular refraction test on an autorefractometer to obtain the best-corrected visual acuity at distance, since the autorefraction accuracy approximates that of the subjective refraction [2, 3]. However, objective ocular refraction differs to some degree from subjective ocular refraction. This is because an autorefractometer evaluates the objective ocular refraction based on corneal and lens power and axial length [4], whereas subjective ocular refraction tests include other elements, such as depth of focus [5], cone density, and visual processing [6, 7]. 

In addition, subjective refraction aims to provide the most comfortable vision in daily life because different individuals use eyeglasses in different situations, such as using a computer [8], reading [9], and may prefer correction optimized to attain certain goals such as the prevention of progression of myopia [10]. Moreover, the subjective ocular refraction test in clinics generally examines the eyes individually, whereas individuals use their eyes in a binocular fashion. Ocular refraction under binocular conditions was also shown to be more myopic than that under monocular conditions, owing to convergence accommodation under binocular conditions [11]. Thus, the examiners may test the eyes under binocular conditions to provide the best refractive correction under these conditions. However, conventional refractive tests cannot simultaneously confirm the ocular refraction in either eye. Thus, the lack of binocular refraction methods may cause some patients to feel discomfort while wearing their eyeglasses [12]. 

The Chronos binocular/monocular refraction system (Chronos; Topcon Corp., Tokyo, Japan) allows objective and subjective refraction under binocular (binocular mode) and monocular (monocular mode) conditions to be performed on a single instrument and has been developed to overcome these issues. Objective and subjective ocular refraction tests using Chronos were designed to maintain binocular vision because the subjects were simultaneously looking through the left and right lens barrels with both eyes. Furthermore, ocular refractive tests under binocular vision are expected to be more relevant to daily vision than conventional refractive tests under monocular conditions.

However, the consistency between binocular testing using the Chronos binocular/monocular refraction system and conventional monocular refractive examination is unknown, and differences from conventional methods must be clearly defined if the Chronos binocular/monocular refraction system is to be used clinically. Therefore, this study aimed to compare the Chronos binocular/monocular refraction system with conventional methods of objective ocular refraction using a conventional autorefractometer, and subjective ocular refraction using a trial-frame in real space.

## Methods

### Participants

In total, 28 healthy volunteers (mean ± standard deviation, 21.2 ± 1.5 years old; range: 18–25 years) were included in this prospective study. All subjects underwent complete ophthalmological examinations, including measures of best-corrected distance visual acuity at 5.0 m, stereoacuity at 40 cm (Titmus Stereotest; Stereo Optical Co., Inc., Chicago, USA), close (33 cm) and distant (5.0 m) alternating prism cover tests to identify heterophoria, and fundus examinations. Patients with ocular disease and a history of ocular surgery were excluded from the study. The individuals with large phorias were not excluded in this study.

After explaining the nature of the study and possible complications, all subjects provided informed consent for inclusion in the identification of information/images in an online open-access publication. This study adhered to the tenets of the 2013 revised Declaration of Helsinki by the World Medical Association. The Institutional Review Board of Teikyo University approved the experimental protocol and consent procedures (approval no. 21–067).

### Apparatus

#### Chronos

**Fig. 1:**
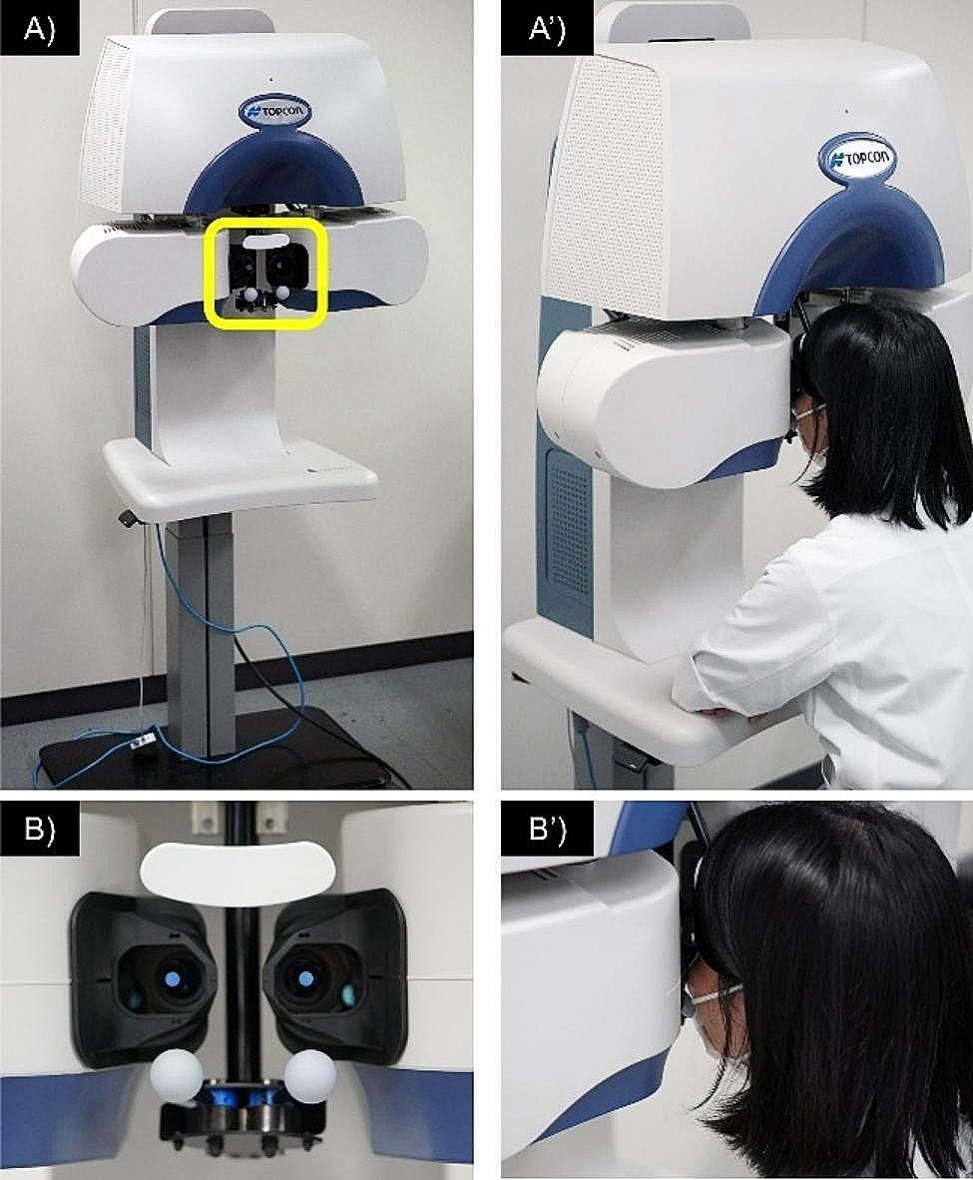
Experimental Chronos binocular/monocular refraction system set up. (**B**) Enhanced image of the yellow square in (**A**). (**A**’) and (**B**’) show the images acquired during the actual measurement. A headrest and cheek rest to fix the participant’s head are visible

This study used the Chronos binocular/monocular refraction system (Fig. 1). It was equipped with two auto refractometers based on the KR-800 automated refractometer (Topcon Corp, Tokyo, Japan) to measure the objective ocular refraction from − 25.00 to + 22.00 diopters (D) for spherical refractive error and up to 10.00 D of cylinder refractive error. The eye trackers detected each eye from the pupil and corneal reflections, positioned the auto-refractometers in front of each eye, and measured the ocular refraction in both eyes. The target fixation image was a house with a red roof.

The Chronos binocular/monocular refraction system uses several optical display systems for subjective refraction. The two optical systems project liquid-crystal images onto the subject’s right and left eyes and use the following four mechanisms to achieve a subjective refraction function:

1. Control of the convergence stimulus: The angle of measurement is adjusted by the three motors to realise a convergence stimulus at any distance from 25 cm to 6 m.
2. Control of accommodation stimulus: The spherical lens position is adjusted using one motor to realise accommodation stimuli at any distance from 25 cm to 6 m.
3. Control of spherical power: Spherical lens position is adjusted by one motor to realise any spherical power setting from + 18D to -18D.
4. Control of cylindrical power and cylindrical axis: The rotation angle of the two cylindrical lenses is adjusted by two motors to realise any cylindrical power and cylindrical axis setting from 0D to -8D [13, 14].

**Fig. 2:**
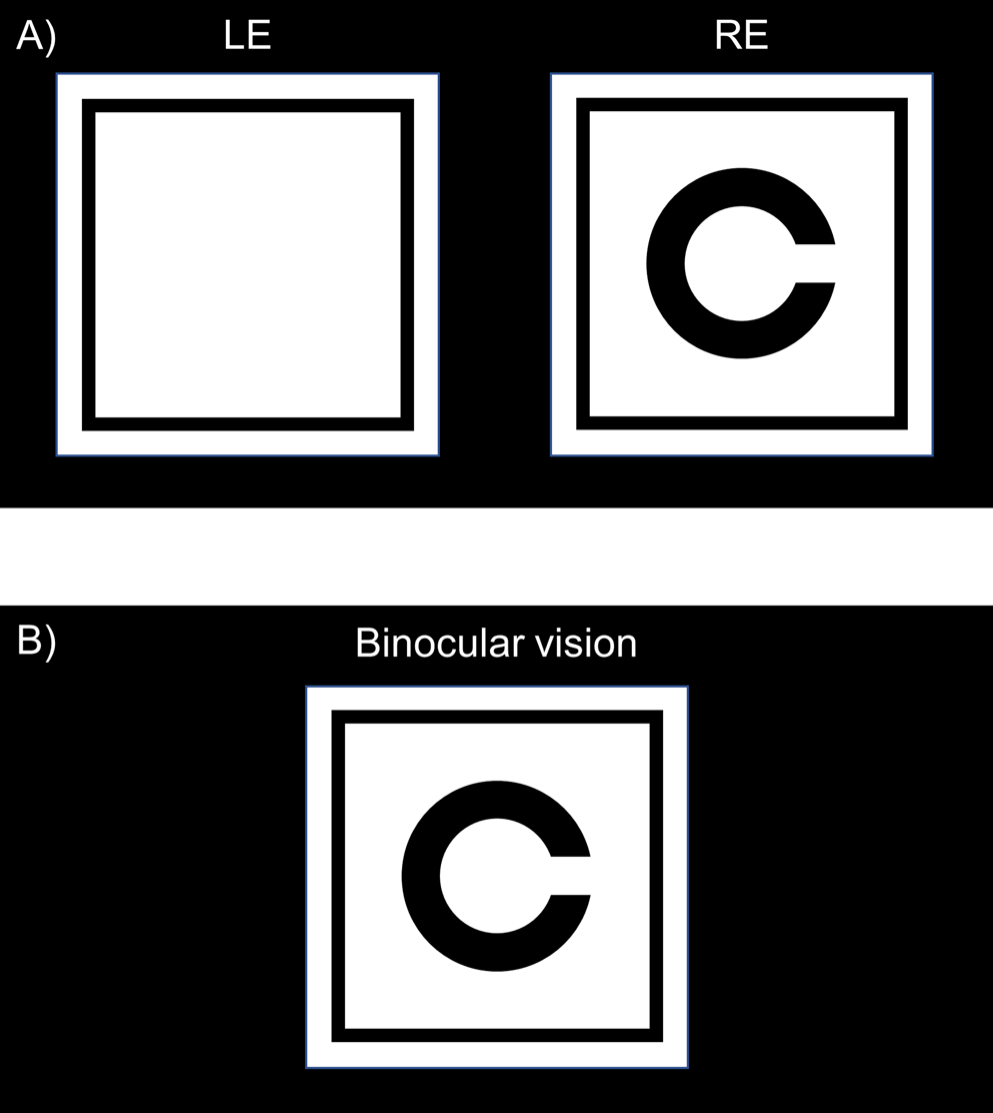
Participant’s image displays during a subjective refraction test using Chronos binocular/monocular refraction system. The Chronos binocular/monocular refraction system displays the target in one eye. However, the peripheral black frame remains (**A**) to maintain binocular fusion (**B**). LE, left eye; RE, right eye

Chronos binocular/monocular refraction system can display optotypes on the two liquid crystal displays and occlude a non-tested eye using the console; however, the display for that eye does not turn off completely. The target (e.g. the Landolt ring) vanishes (Fig. 2A), whereas the peripheral frame remains to maintain binocular fusion (Fig. 2B).

**Fig. 3:**
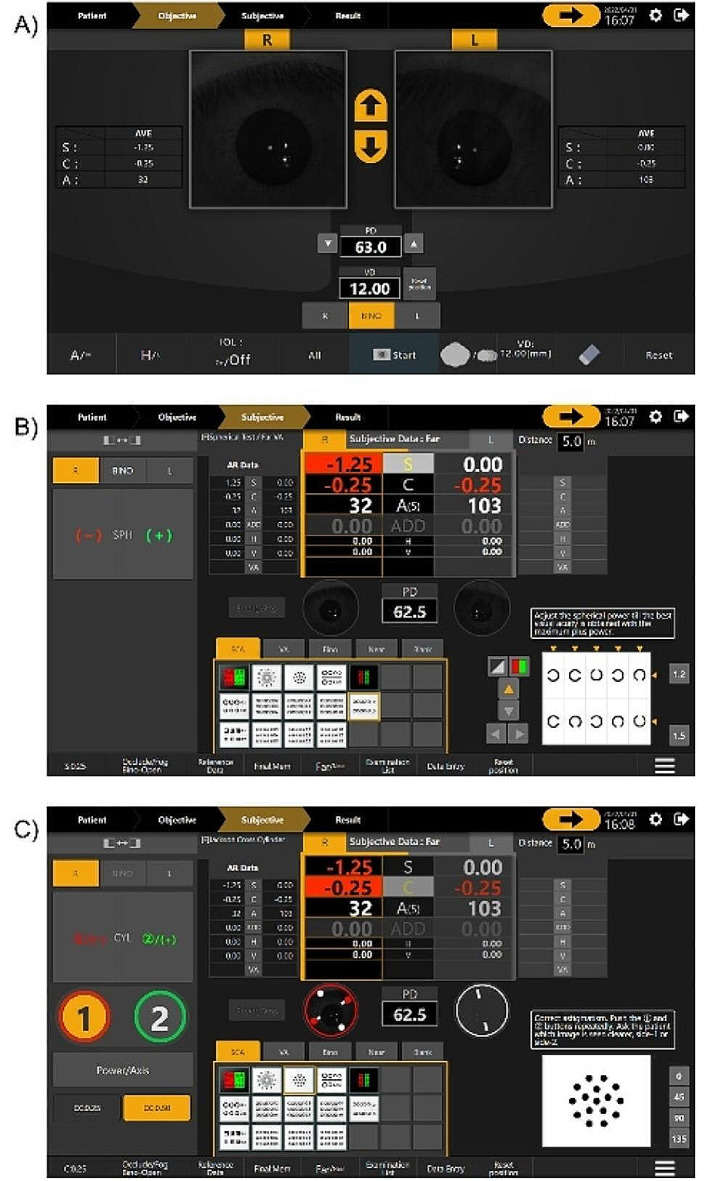
Control screen in the examination using Chronos binocular/monocular refraction system. Pressing the start panel initiated the objective refractive test in both eyes (**A**). This was followed by the subjective refraction test (**B**). The cylindrical lens was adjusted using a Jackson cross cylinder (**C**). In (**A**), S, C, A, PD, and VD indicate spherical power, cylindrical power, cylindrical axis, pupillary distance, and vertex distance, respectively. AVE indicates the average values of three measurements for the spherical lens power, cylindrical lens power, and cylindrical axis. (**B**) BINO, R, and L indicate binocular and monocular vision in the right and left eyes, respectively. The initial lens power in the subjective refraction test corresponds to the autorefractometer values in the AR Data. SPH is the spherical lens power. In (**C**), CYL and CC indicate the cylindrical lens and the cross-cylinder, respectively

The subjects fixated on the Landolt ring at a 5.0 m distance, which was adjusted optically, and the first ophthalmic lenses were selected automatically, based on the objective ocular refraction in both eyes. The examiner determined the subjective ocular refraction while examining the participant’s response using a Bluetooth tablet linked to Chronos binocular/monocular refraction system. The tablet device can change the status of the Chronos binocular/monocular refraction system, such as the measuring distance, spherical and cylindrical lens power, and target (Fig. 3).

#### Conventional auto refractometer

A KR-800 refractometer was used as the conventional autorefractometer. It can measure monocular objective ocular refraction over the same range as that measured by Chronos binocular/monocular refraction system.

### Subjective ocular refraction

**Fig. 4:**
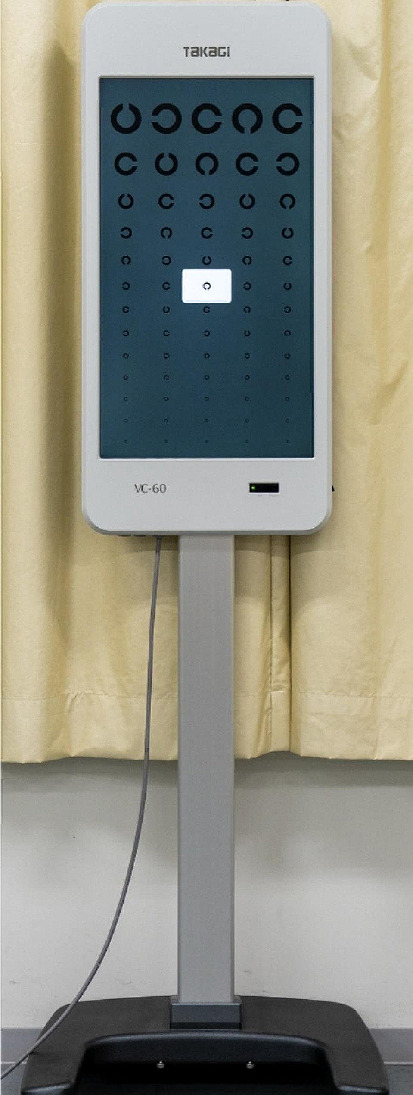
Exterior of VC-60 VC60 is an optotype on an LCD display. The subjects were required to indicate the direction of the notch in the presented Landolt ring

Subjective ocular refraction tests were performed in a well-lit room (600 lx), with both Chronos binocular/monocular refraction system and the trial-frame in real space using VC-60 (Takagi Co., Ltd., Nagano, Japan; Fig. 4), a standard visual acuity chart. The examiner presented the Landolt ring on a liquid-crystal display (resolution of 2560 × 1440 pixels), corresponding to the controller from a distance. The luminance of the backlight was 300 cd/m^2^.

Subjective ocular refraction was determined by combining the maximum plus or minimum minus spherical and cylindrical lenses necessary to provide a best-corrected visual acuity of − 0.176 logMAR (logarithm of the minimum angle of resolution) at an optical and natural distance of 5.0 m (with decimal acuity of 1.5).

The initial spherical lens was determined by adding + 1.00 D to the objective ocular refraction to prevent overcorrection, and the examiner (M.F.) confirmed that the participant was unable to clearly see the − 0.176 logMAR optotype with this lens. The examiner then added spherical lenses in increments of − 0.25D to obtain the highest visual acuity with spherical lenses alone. Additionally, the examiner used Jackson’s Cross Cylinder of ± 0.50 D and ± 0.25 D to determine the cylindrical lens after the single spherical lens obtained the highest visual acuity. Dot targets were used to determine the power and axes of the cylindrical lenses. The cylindrical lens axis was determined at 5° increments. After identifying the cylindrical lens, the examiner readjusted the spherical lens.

The procedure for subjective refraction test was as follows.

1. First, the subjective lens was selected by referring to the objective ocular refraction of both eyes. For the spherical lens, a + 1D lens was added to prevent overcorrection.
2. Showing the Landolt ring on the display, spherical lenses were added so that the subjects could obtain the highest visual acuity.
3. Showing dot targets, cylindrical lenses and axis were corrected using Jackson’s Cross Cylinder of ± 0.50 D and ± 0.25 D. The cylindrical lenses were added, and the axis was determined using 5° increments.
4. Showing the Landolt ring, spherical lenses were recorded so that the subjects could obtain their best-corrected visual acuity.

The procedure was the same for the subjective refraction test using Chronos binocular/monocular refraction system and the trial-frame in real space. However, the test using the Chronos binocular/monocular refraction system did not examine the peripheral frame of the eye to maintain binocular fusion. Although binocular balance is important in clinical practice, only the monocular visual acuity was evaluated in this study.

**Fig. 5:**
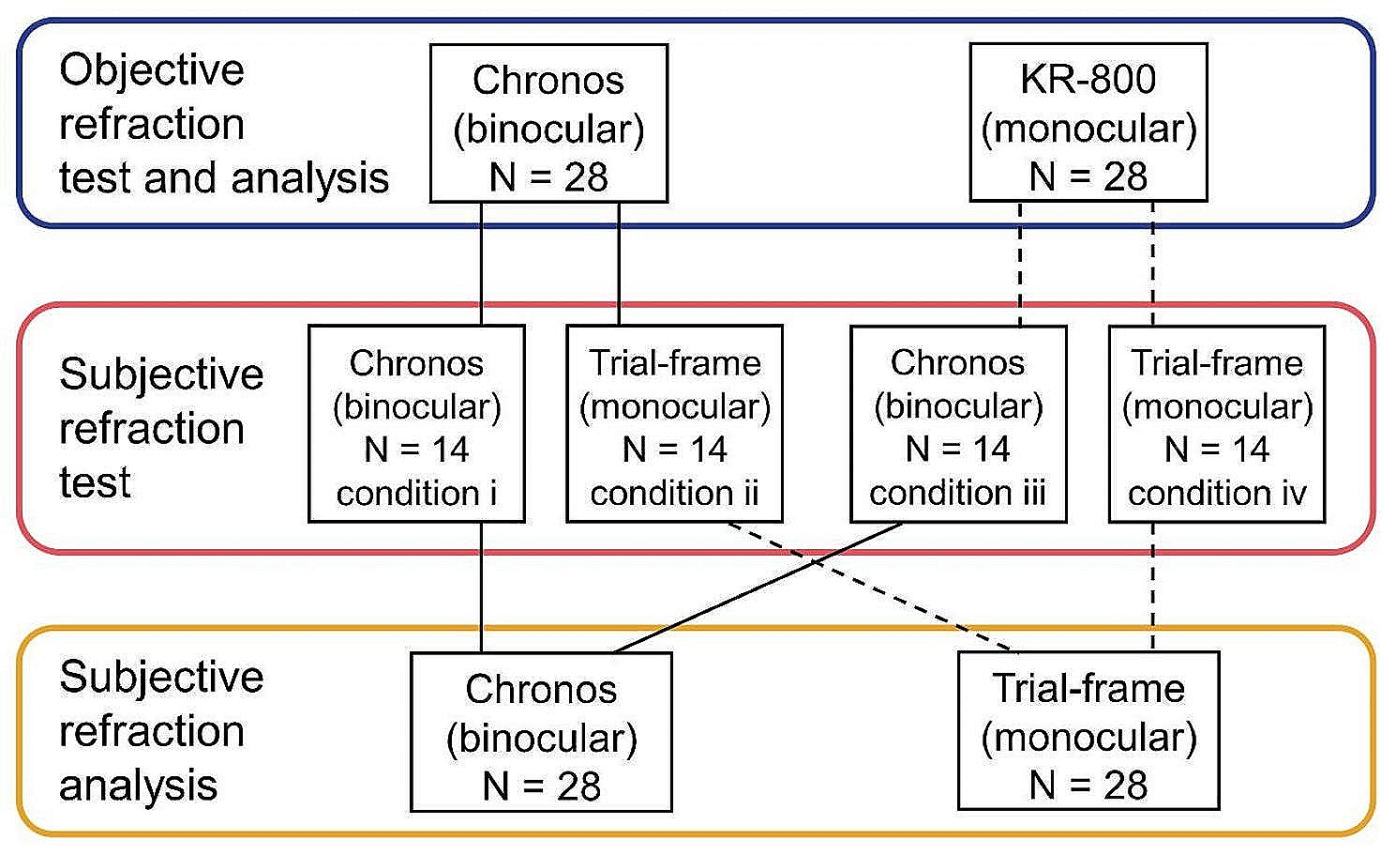
Examination flow The solid and dashed lines indicate binocular and monocular conditions, respectively. The subjective refractive test in the trial-frame was performed in real space. Chronos, Chronos binocular/monocular refraction system; Trial-frame, Trial-frame in real space

We were concerned that the results of the subjective refractive test would be affected by the objective ocular refraction results. Therefore, the subjects underwent the visual acuity test four times under four random conditions, as follows Fig. 5.

### Additional experiment: Chronos binocular/monocular refraction system under monocular vs. binocular conditions

As the default setting of the Chronos binocular/monocular refraction system is to test with both eyes open, it does not entirely match the conditions of a conventional subjective ocular refraction test conducted with a single eye separately. Thus, we conducted the subjective ocular refraction test on Chronos binocular/monocular refraction system under monocular conditions, such as conventional subjective ocular refraction, with one eye occluded.

The subject’s left eye was completely occluded with white gauze (Surgical Pad, Hakujuji Co., Ltd.) to assess the difference between the binocular and monocular conditions in the Chronos binocular/monocular refraction system. Subjective refraction in the additional experiment was initiated by adding + 1.00 D from the subjective spherical equivalents (SE) of the previous subjective ocular refraction test. The experimental environment and procedures for the Chronos binocular/monocular refraction system were the same as those used in the previous experiments.

### Data acquisition conditions

In this study, the ocular refraction data were obtained under five conditions　(Fig. 5; Table 1).

**Table 1:** Conditions in the objective and subjective refraction tests

	Objective testing	Subjective testing
Test unit (Chronos binocular/monocular refraction system)	Binocular conditions	• Binocular conditions • Monocular conditions
Controls (monocular conditions)	Conventional autorefractometer (KR-800)	Trial-frame refraction (“real space”)

The objective ocular refraction was measured using two tests: the Chronos binocular/monocular refraction system under binocular conditions and a conventional autorefractometer under monocular conditions.

The subjective ocular refraction was measured using three tests: Chronos binocular/monocular refraction system under binocular and monocular conditions and trial-frame in the real space under monocular conditions.

### Data analysis

The objective and subjective ocular refractions were converted to SEs as follows:

$$ SE = S+\frac{C}{2}$$

where *S* and C are the spherical and cylindrical lens power, respectively. In this study, a cylindrical lens was used only in the negative format.

Objective and subjective astigmatism were converted from spherocylindrical to power vector notation by applying Fourier transformation using the following equations:

$$ {J}_{0}= -\frac{C}{2}\times cos2a$$

$$ {J}_{45}= -\frac{C}{2}\times sin2a$$

where *α* is an axis of a cylindrical lens, *J*_*0*_ indicates the cylinder lens power set at 90° and 180°, the positive values of *J*_*0*_ were with the rule (WTR) astigmatism, the negative values of J_0_ were against the rule astigmatism, and *J*_*45*_ is the cylinder lens power set at 45° and 135°, representing oblique astigmatism.

The simple conversion from the power vector notation to conventional notation is twice the square root of the sum of *J*_*0*_^*2*^ and *J*_*45*_^*2*^.

### Statistical analysis

Bland–Altman analysis was performed to compare the objective ocular refractions in SE using *J*_*0*_ and *J*_*45*_ of the Chronos binocular/monocular refraction system under binocular conditions and KR-800 under monocular conditions, as well as the subjective ocular refractions in SE using *J*_*0*_ and *J*_*45*_ of the Chronos binocular/monocular refraction system under binocular conditions and the trial-frame in real space under monocular conditions.

After assessing the normality of variable distributions using Shapiro–Wilk tests, the fixed and proportional biases between the two apparatuses and the measurement conditions were analysed using paired t-tests and single linear regression analyses.

The chronosinocular/monocular refraction system maintains the binocular fusion. Therefore, the influence of fusional convergence was considered. The difference in the objective ocular refraction between the Chronos binocular/monocular refraction system under binocular conditions and the KR-800 under monocular conditions was calculated because of accommodation by convergence. The relationship between the degree of heterophoria and the differences in objective ocular refraction was analysed using Spearman’s rank correlation coefficients.

In an additional experiment, the differences between the Chronos binocular/monocular refraction system under monocular and binocular conditions and real space under monocular conditions were analysed using a paired t-test following the assessment of normality distribution using the Shapiro–Wilk test with Bonferroni correction.

**Fig. 6:**
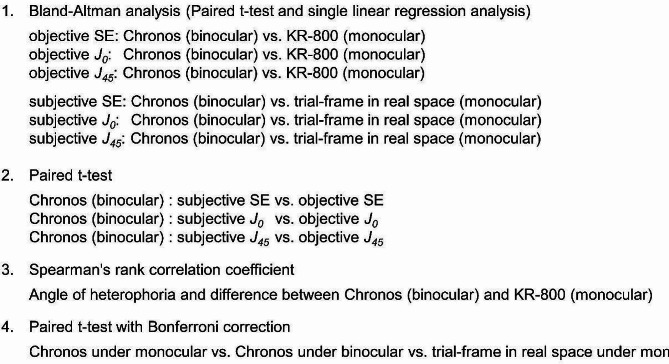
Statistical Analysis Items

IBM SPSS Statistics for Windows version 26 (IBM Corp., Armonk, NY, USA) was used to determine the significance of the differences, and *P* < 0.05 was considered statistically significant (Fig. 6).

This study achieved five ocular refraction results: two objective ocular refractions of the Chronos binocular/monocular refraction system under binocular conditions and KR-800 under monocular conditions, three subjective ocular refractions of the Chronos binocular/monocular refraction system under binocular conditions, monocular conditions, and a trial-frame in real space under monocular conditions. Bland–Altman analysis was performed to analyse the consistency of the Chronos binocular/monocular refraction system under binocular conditions and the conventional method, which measures monocularly, followed by evaluation of the difference between objective and subjective ocular refraction in the Chronos binocular/monocular refraction system under binocular conditions. The Spearman’s rank correlation coefficient was used to evaluate the correlation between objective ocular refraction by fusional convergence in the Chronos binocular/monocular refraction system under binocular conditions and KR-800 under monocular conditions. Finally, a paired t-test with Bonferroni correction was conducted to assess whether the subjective ocular refraction in the Chronos binocular/monocular refraction system under monocular conditions differed from the Chronos binocular/monocular refraction system under binocular conditions and the real space under monocular conditions.

## Results

### **Characteristics of the subjects**

The participants’ characteristics are listed in Table 2. The objective refraction was − 3.80 ± 2.68 D using the KR-800 under monocular condition. The angle of heterophoria was − 6.9 ± 5.9 prism diopter (PD) at near distance and − 3.3 ± 4.3 PD at far distance. All subjects had stereo acuity of 1.60 log arcsec (40 s equally).

**Table 2:** Characteristics of the subjects

Subject	Age (years)	Sph (D)	Cyl (D)	Ax (°)	APCT (PD)		Stereo acuity	PD (mm)
					Near	Distant		
S1	23	−6.50	0	180	−16	−16	40	62.5
S2	21	−1.50	0	180	−2	−2	40	58
S3	21	−1.75	−1.00	90	−8	−4	40	61.5
S4	20	−0.50	−0.25	70	−16	−6	40	61
S5	21	−2.25	−0.50	170	−4	0	40	65.5
S6	21	−7.50	−3.00	175	−20	−14	40	61
S7	19	−4.75	−2.00	175	−6	0	40	62
S8	20	−4.00	−0.75	160	−12	−6	40	66.5
S9	23	−7.75	−1.00	180	−8	0	40	61
S10	25	−2.50	−0.50	145	1	−2	40	62
S11	21	−7.75	−1.00	175	−14	−8	40	59.5
S12	22	−1.75	0	180	2	−2	40	57.5
S13	21	−6.25	−1.00	170	−8	−4	40	61
S14	18	−5.25	−0.25	30	−12	−2	40	64.5
S15	19	−5.50	−0.75	165	−4	−1	40	61.5
S16	22	0	0	180	−4	0	40	65
S17	22	−1.00	−0.25	100	−6	−2	40	59
S18	21	−4.75	−0.75	175	−20	−12	40	67
S19	21	−3.75	−1.50	180	−4	0	40	59.5
S20	21	−0.75	−0.25	115	−2	−2	40	58
S21	24	0.25	−0.25	180	−8	−4	40	61
S22	21	−0.75	−0.75	160	0	0	40	58
S23	21	−5.25	−0.75	170	−6	0	40	69.5
S24	23	−4.50	−1.75	175	−2	−2	40	62.5
S25	22	−4.75	−0.50	165	−4	−2	40	61
S26	23	−0.75	−0.25	155	0	0	40	59.5
S27	21	−5.00	−1.25	175	−4	−2	40	60.5
S28	23	−1.00	−0.50	5	−2	0	40	62.5

Sph, spherical power; Cyl, cylindrical power; Ax, cylindrical axis; APCT, alternative prism cover test; PD, pupillary distance

### Objective ocular refraction: Chronos binocular/monocular refraction system under binocular condition) vs. KR-800 under monocular condition

The objective ocular refraction test in the Chronos binocular/monocular refraction system defaults to binocular conditions. Therefore, objective ocular refraction may differ from that of the conventional autorefractometer performed under monocular conditions. Therefore, we compared the objective ocular refractions of the Chronos binocular/monocular refraction system and KR-800 under binocular and monocular conditions.

**Fig. 7:**
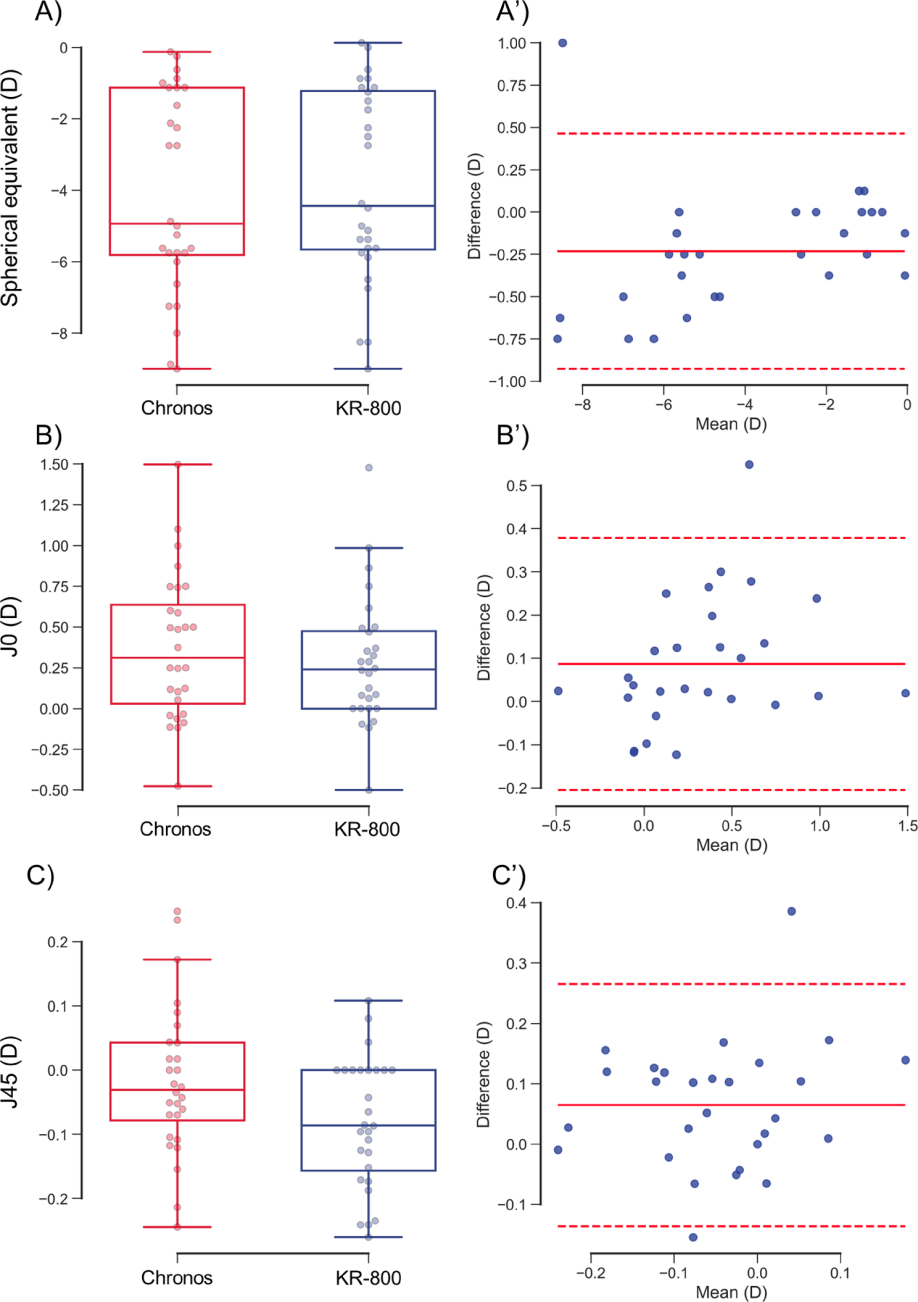
Differences in objective ocular refraction between Chronos binocular/monocular refraction system under binocular condition and KR-800 under monocular condition

**Fig. 8:**
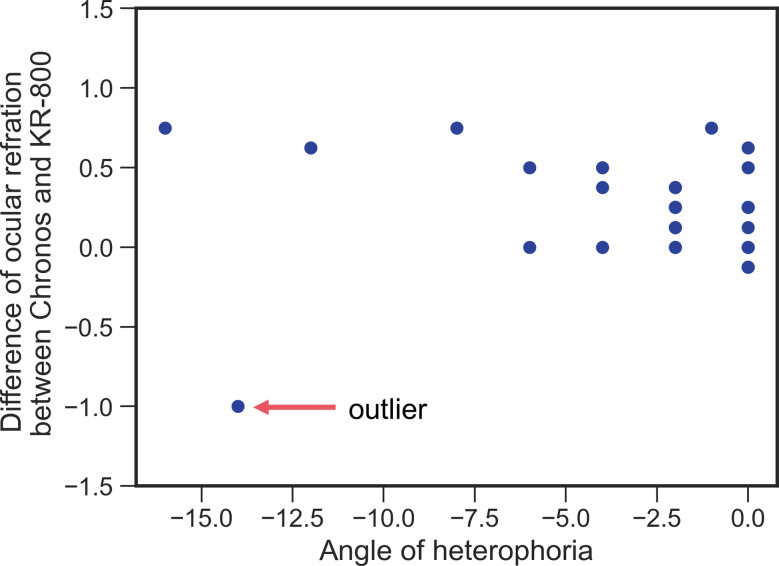
Relationship between Angle of heterophoria and difference of ocular refraction between binocular (Chronos binocular/monocular refraction system) and monocular (KR-800) conditions

The SE obtained with Chronos binocular/monocular refraction system under binocular condition was significantly more myopic (− 4.08 ± 2.76 D) than that of KR-800 under monocular condition (− 3.85 ± 2.66 D) (*P* = 0.002; Fig. 7A; Table 3). No proportional bias was observed between the two autorefractometers (*R*^*2*^ = 0.077, *P* = 0.153; Fig. 7A). The mean value of the differences between Chronos binocular/monocular refraction system under binocular condition and KR-800 under monocular condition was − 0.23 D and the 95% limits of agreement (LOA) ranged from − 0.93 to 0.47 D. The angle of heterophoria was significantly correlated with the difference in SE between the Chronos binocular/monocular refraction system under binocular conditions and KR-800 under monocular conditions without outlier data (without outliers, *rs* = − 0.389, *P* = 0.045; without outliers, *rs* = − 0.231, *P* = 0.163) (Fig. 8).

**Table 3:** Summary in each testing

	Objective testing	Subjective testing
Test unit (Chronos binocular/monocular refraction system)	Binocular conditions: –4.08 ± 2.76 D	• Binocular conditions:–3.55 ± 2.67 D • Monocular conditions:–3.17 ± 2.57 D
Controls (monocular conditions)	Conventional autorefractometer (KR-800): –3.85 ± 2.66 D	Trial-frame in real space –3.33 ± 2.75 D

The *J*_*0*_ of Chronos binocular/monocular refraction system under binocular condition (0.37 ± 0.43 D) was significantly more positive than that of KR-800 under monocular condition (0.29 ± 0.39 D) (*P* = 0.005; Fig. 7B). A proportional bias was not found between the two autorefractometers (*R*^*2*^ = 0.095, *P* = 0.111; Fig. 7B’). The mean value of the differences between Chronos binocular/monocular refraction system under binocular condition and KR-800 under monocular condition was − 0.09 D and the 95% LOA ranged from − 0.20 D − 0.38 D.

The *J*_*45*_ of Chronos binocular/monocular refraction system under binocular condition (− 0.02 ± 0.11 D) was significantly more positive than that of KR-800 under monocular condition (− 0.08 ± 0.10 D) (*P* = 0.003; Fig. 7C). No proportional bias was observed between the two autorefractometers (*R*^*2*^ = 0.029, *P* = 0.385; Fig. 7C). The mean value of the differences between Chronos binocular/monocular refraction system under binocular condition and KR-800 under monocular condition was − 0.06 D and the 95% LOA ranged from − 0.14 D − 0.26 D.

Red and blue boxplots with dots indicate the objective SE in the Chronos binocular/monocular refraction system and the KR-800 (A–C). The solid and dashed red lines indicate the average and 95% limits of agreement in the differences between Chronos binocular/monocular refraction system and KR-800 (A’–C’). The SE (A), J_0_ (B), and J_45_ (C) differed significantly between the two systems and KR-800. No proportional bias was observed between the two devices.

The angle of heterophoria was not significantly correlated with difference of SE between Chronos binocular/monocular refraction system and KR-800 without outlier (*rs* = − 0.389, *P* = 0.045) and with outlier (*rs* = − 0.231, *P* = 0.163).

### Subjective refraction test: Chronos binocular/monocular refraction system under binocular condition vs. conventional trial-frame real space under monocular condition

The subjective ocular refraction test in the Chronos Binocular/Monocular Refraction System defaults to binocular conditions. A Landolt ring was presented to the subject’s eye, and a blank was shown to the other eye. However, black frames that maintain binocular fusion remain in both eyes. Therefore, subjective ocular refraction between the Chronos binocular/monocular refraction system under binocular conditions and the trial-frame in real space under monocular conditions may differ.

**Fig. 9:**
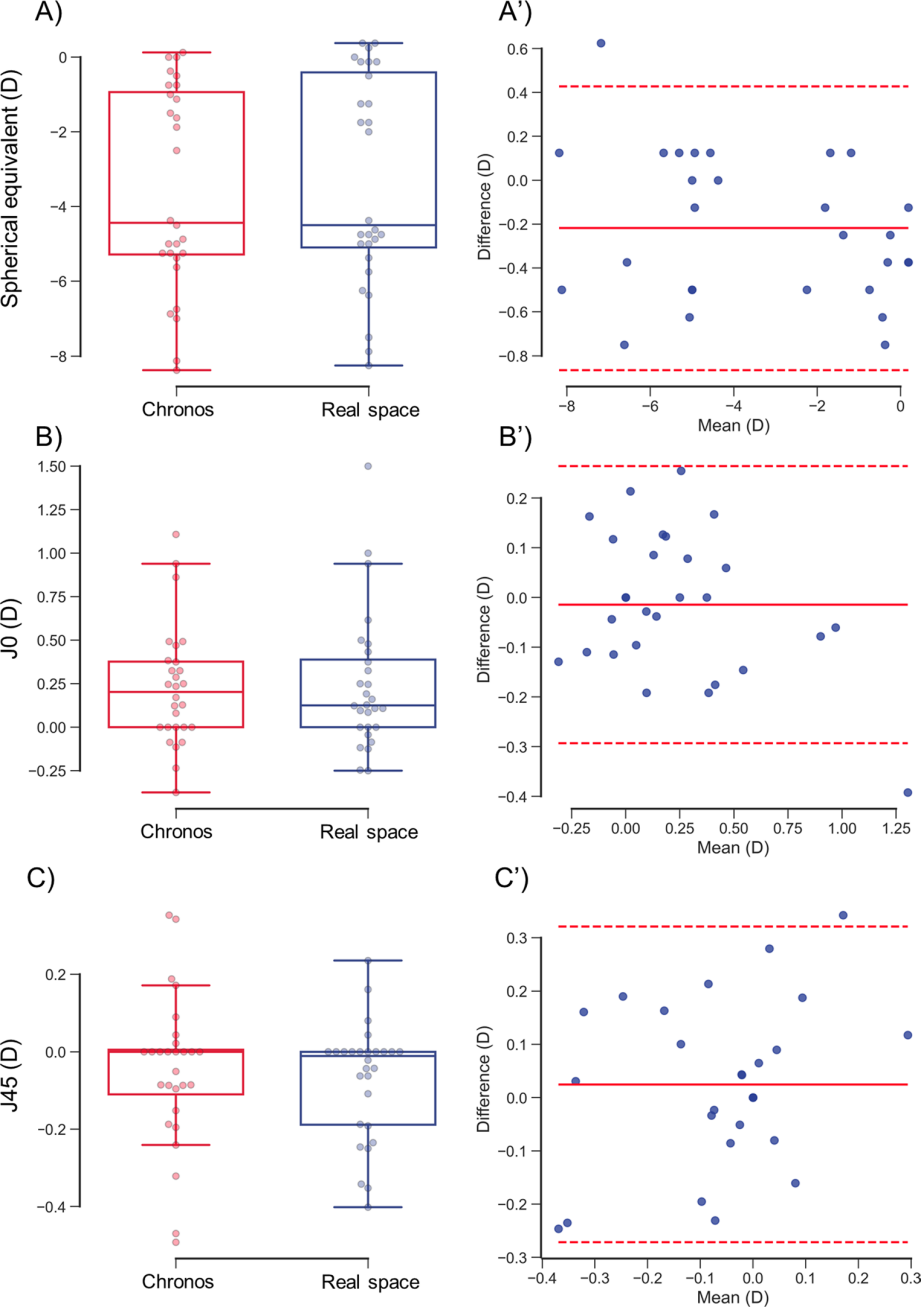
Subjective refraction between Chronos binocular/monocular refraction system under binocular condition and trial-frame in real space under monocular condition

The subjective SE from Chronos binocular/monocular refraction system under binocular condition (− 3.55 ± 2.67 D) was significantly more myopic than that from trial-frame in real space under monocular condition (− 3.33 ± 2.75 D) (*P* = 0.002; Fig. 9A; Table 3). No proportional bias was observed between the two examinations (*R*^*2*^ = 0.070, *P* = 0.174; Fig. 9A). The mean value of the differences between the Chronos binocular/monocular refraction system under binocular conditions and the trial-frame in real space under monocular condition was − 0.22 D. The 95% LOA ranged from − 0.87 − 0.43 D.

The *J*_*0*_ was not significantly different between Chronos binocular/monocular refraction system under binocular condition (0.23 ± 0.34 D) and trial-frame in real space under monocular condition (0.24 ± 0.39 D) (*P* = 0.61; Fig. 9B). No proportional bias was observed between the two examinations (*R*^*2*^ = 0.137, *P* = 0.053; Fig. 9B). The mean value of the differences between Chronos binocular/monocular refraction system under binocular condition and trial-frame in real space under monocular condition was − 0.01 D, and the 95% LOA ranged from − 0.29 − 0.26 D.

The *J*_*45*_ was not significantly different between Chronos binocular/monocular refraction system under binocular condition (− 0.05 ± 0.19 D) and trial-frame in real space under monocular condition (− 0.07 ± 0.15 D) (*P* = 0.41; Fig. 9C). No proportional bias was observed between the two examinations (*R*^*2*^ = 0.093, *P* = 0.115; Fig. 9C). The mean value of the differences between the Chronos binocular/monocular refraction system under binocular conditions and the trial-frame in real space under monocular condition was 0.02 D, and the 95% LOA ranged from − 0.27 − 0.32 D.

The red and blue boxplots with dots indicate the objective SE in the Chronos binocular/monocular refraction system under binocular conditions, and the trial-frame in real space under monocular conditions (A–C). The solid and dashed red lines indicate the average and 95% limits of agreement of the difference between the Chronos binocular/monocular refraction system under binocular conditions and the trial-frame in real space under monocular conditions (A’–C’). Only SE (A) was significantly and negatively greater in the Chronos binocular/monocular refraction system than in the trial-frame in real space. No proportional bias is observed between the two devices.

### Difference between objective and subjective chronos test

In general, objective ocular refraction measured by autorefractometer is more negative than subjective ocular refraction [15]. We examined whether similar results occurred in the Chronos binocular/monocular refraction system.

The SE and *J*_*0*_ were significantly more myopic in the objective test (SE, − 4.08 ± 2.76 D; *J*_*0*_, 0.37 ± 0.43 D) than in the subjective test (SE, − 3.55 ± 2.67 D; *J*_*0*_, 0.23 ± 0.34 D) in Chronos binocular/monocular refraction system under binocular condition (*P* < 0.001). *J*_*45*_ did not significantly differ between the objective (− 0.02 ± 0.11 D) and subjective (− 0.05 ± 0.19 D) tests under binocular condition (*P* = 0.145).

### Chronos binocular/monocular refraction system under monocular vs. chronos binocular/monocular refraction system under binocular vs. trial-frame in real space under monocular conditions

By default, the Chronos binocular/monocular refraction system performs tests under binocular conditions, which is not the same as the monocular condition used in traditional subjective ocular refraction tests. To ensure accurate results, we conducted a subjective ocular refraction test using the Chronos binocular/monocular refraction system under monocular conditions, similar to a conventional test in which one eye was occluded.

**Fig. 10:**
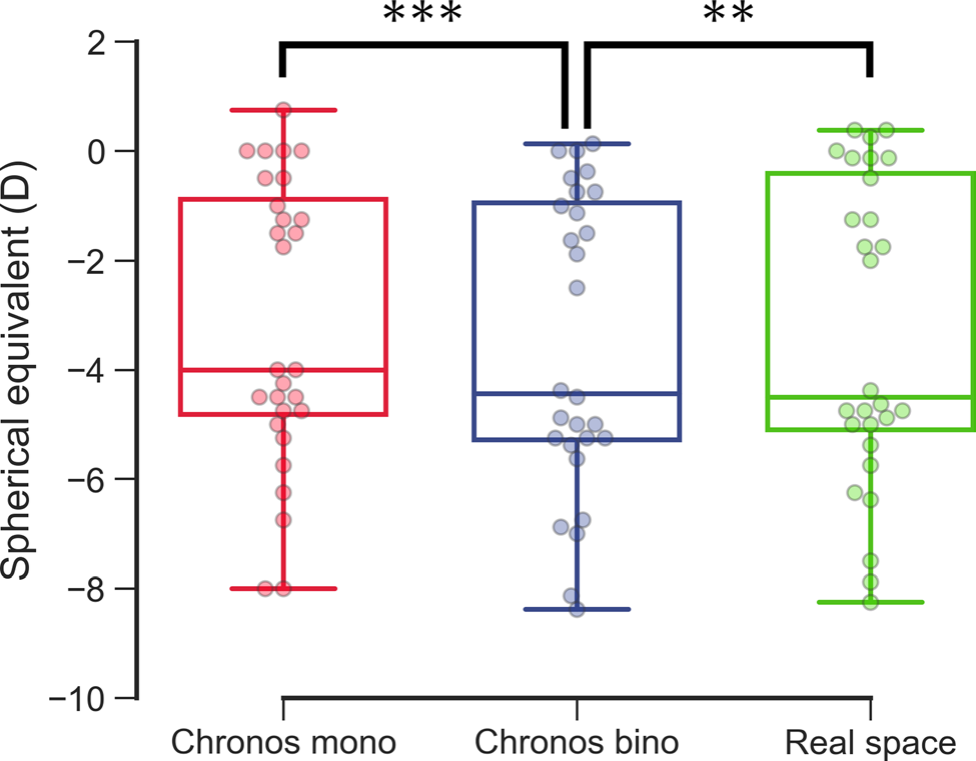
Subjective refraction between Chronos binocular/monocular refraction system under monocular (red) and binocular (blue) conditions and trial-frame in real space under monocular condition (green)

The subjective SE was significantly more hyperopic for Chronos binocular/monocular refraction system under the monocular condition (− 3.17 ± 2.57 D) than under the binocular condition (− 3.55 ± 2.67 D D) (*P* < 0.001; Fig. 9; Table 3). The subjective SE did not significantly differ between Chronos binocular/monocular refraction system under the monocular condition and the real space under monocular condition (− 3.33 ± 2.75 D) (*P* = 0.33; Fig. 10).

The Chronos binocular/monocular refraction system subjective refraction tested under binocular conditions resulted in a significantly more negative spherical equivalent than the Chronos binocular/monocular refraction system subjective refraction tested under monocular conditions and the trial-frame refraction tested in real space and under monocular conditions.

***: *P* < 0.001, Paired t-test with Bonferroni correction.

**: *P* = 0.006, Paired t-test with Bonferroni correction.

## Discussion

### Overview

Objective and subjective ocular refraction were measured using the Chronos binocular/monocular refraction system. The SE in the objective and subjective ocular refractions with Chronos binocular/monocular refraction system under binocular condition changed to myopic values of − 0.23 D and − 0.22 D compared to KR-800 and trial-frame in real space under monocular condition. These findings indicate that the Chronos binocular/monocular refraction system under binocular conditions resulted in refractive errors that were more myopic in both objective and subjective refraction tests under monocular conditions. Therefore, under binocular conditions, the Chronos binocular/monocular refraction system is unsuitable for situations requiring accurate refraction, such as preoperative examinations and eyeglass prescriptions. In contrast, the Chronos binocular/monocular refraction system is suitable for screening under binocular conditions because it can complete both objective and subjective ocular refraction tests in a single unit.

The subjective ocular refraction of the Chronos binocular/monocular refraction system under monocular conditions was significantly more hyperopic than that under binocular conditions, and was not significantly different from that of the trial-frame in real space under monocular conditions. These findings suggest that subjective ocular refraction in the monocular condition provides less myopia results than the binocular condition, and that the monocular condition results are comparable to the trial-frame in real space.

### Objective ocular refraction

The Chronos binocular/monocular refraction system was equipped with two automated refractometers based on a KR-800 conventional autorefractometer. However, the objective SE was significantly more myopic with the Chronos binocular/monocular refraction system under binocular conditions than with the KR-800 under monocular conditions (Fig. 7A and A’). The subjects maintained binocular fusion using Chronos to measure objective ocular refraction. Conversely, the KR-800 measures objective ocular refraction without fusional convergence because it uses monocular measurements, and convergence induces accommodation [11]. Hence, we considered accommodation to be induced by fusional convergence by using Chronos.

For objective astigmatism, *J*_*0*_ and *J*_*45*_ were significantly more positive with the Chronos binocular/monocular refraction system under binocular conditions than with the KR-800 under monocular conditions (Fig. 7B and B’, 7 C, and 7 C’). An increase in *J*_*0*_ indicates that the WTR becomes stronger, and an increase in *J*_*45*_ indicates that the power of the ocular refraction shifts from 135° to 45°. Our findings are consistent with those of Porrill et al., who demonstrated that both eyes were inverted during convergence [16]. These findings support our hypothesis that accommodation is induced by fusional convergence, using the Chronos binocular/monocular refraction system. However, previous studies reported that accommodation alters astigmatism [17]. Therefore, measuring the amplitude of fusional convergence may be necessary to test our hypothesis in future studies.

### Subjective ocular refraction

Regarding subjective ocular refraction, the subjective SE obtained with the Chronos binocular/monocular refraction system under binocular condition was significantly more myopic than that obtained trial-frame in real space under monocular condition (Fig. 9A and A′). We believe that subjective ocular refraction under binocular conditions shifted to myopia owing to accommodation related to convergence. Therefore, the results of the subjective ocular refraction test may depend on those of the objective ocular refraction test [18]. To minimize the bias of the objective ocular refraction in the subjective ocular refraction test, we mixed the objective ocular refraction measured by the Chronos binocular/monocular refraction system under binocular condition and KR-800 under the monocular condition. In this study, the measurement distance of subjective ocular refraction was the same between the Chronos binocular/monocular refraction system under binocular conditions and the trial-frame in real space under monocular conditions.

To maintain binocular fusion, the peripheral frame of the eye was not examined in the subjective refraction test using the Chronos binocular/monocular refraction system under binocular conditions (Fig. 3). We occluded the subject’s left eye to avoid binocular fusion when using the Chronos binocular/monocular refraction system in an additional experiment. The subjective SE under monocular conditions showed a hyperopic shift relative to that under binocular conditions. Furthermore, the subjective SE did not significantly differ between the Chronos binocular/monocular refraction system under monocular conditions and the trial-frame in real space under monocular conditions (Fig. 10). These findings support our hypothesis that convergence accommodation through fusional convergence works by using a Chronos binocular/monocular refraction system under binocular conditions. Moreover, with the Chronos binocular/monocular refraction system, an image from one eye can be removed via the console. Therefore, we can set the condition of the subjective refraction test within an error of 0.25 D between the conventional monocular test and the binocular test as required, which is closer to the trial-frame in real-space vision.

However, the subjective *J*_*0*_ and *J*_*45*_ were not significantly different between the Chronos binocular/monocular refraction system under binocular conditions and the trial-frame in real space under monocular conditions (Fig. 9A and A’, 9B, and 9B’). We assume this was due to Jackson’s cross-cylinder method. Moreover, the astigmatism axis was determined in 5° increments. For example, if the axis of astigmatism is changed from 0° to 5° and the astigmatism power is increased by − 0.25 D, *J*_*0*_ changes by 0.11 D. Although the incyclodeviation (this is intorsion) also occurs during the subjective refraction test similar with the objective refraction test, Chronos binocular/monocular refraction system under binocular condition can determine subjective ocular refraction with similar accuracy as the trial-frame in real space under monocular condition, if the astigmatic axis changed in 5° increments in Jackson’s Cross Cylinder.

In this study, we compared objective and subjective ocular refractions in the Chronos binocular/monocular refraction system under binocular and monocular conditions and a trial-frame in real space under monocular conditions; however, eye movements during the examinations could not be evaluated. Particularly, the results of the present study suggest that fusional convergence occurs under binocular conditions when using Chronos binocular/monocular refraction system (Fig. 8). Furthermore, there were no participants with esophoria, despite the random recruitment of participants in this study. Therefore, to test our hypothesis, it was necessary to simultaneously measure eye movements during ocular refraction tests under non-cycloplegic and cycloplegic conditions to confirm whether fusional convergence is induced by the peripheral frame. Thus, in future studies, we aim to include an eye tracking system to Chronos binocular/monocular refraction system to assess the consistency of eye movements and ocular refraction upon recruiting subjects with esophoria, exophoria, and orthophoria.

## Conclusion

The objective and subjective SEs were significantly more negative when using the Chronos binocular/monocular refraction system under binocular conditions than when using the trial-frame in real space under monocular conditions. The subjective SE under monocular conditions using the Chronos binocular/monocular refraction system was comparable to that of a trial-frame in real space under monocular conditions. These findings suggest that the Chronos binocular/monocular refraction system, capable of completing objective and subjective ocular refraction testing with a single unit, is suitable for screening ocular refraction, although it produces slightly more myopic results. Furthermore, the subjective ocular refraction testing accuracy in the Chronos binocular/monocular refraction system can be made equivalent to a trial-frame in real-space testing by switching from binocular to monocular conditions.

## Data Availability

All data related to this study are stated in the paper.

## Abbreviations

SE: spherical equivalents
D: diopters
logMAR: logarithm of the minimum angle of resolution
WTR: with the rule
LOA: limits of agreement
